# “What about Military Decision-Making?”: A Bibliometric Review of Published Articles

**DOI:** 10.3390/bs14070514

**Published:** 2024-06-21

**Authors:** Ivan D’Alessio, Umberto Aitella, Anna Maria Giannini, Jessica Burrai

**Affiliations:** Department of Psychology, Faculty of Medicine and Psychology, Sapienza University of Rome, 00185 Rome, Italy; aitella.1712631@studenti.uniroma1.it (U.A.); annamaria.giannini@uniroma1.it (A.M.G.); jessica.burrai@uniroma1.it (J.B.)

**Keywords:** military decision-making, bibliometric analysis, military decision-making review, defence decision-making analysis, army decision making bibliometric review

## Abstract

Decision-making processes in the military domain constitute a strategic field of research in cognitive psychology, although there are currently few scientific publications addressing the topic. Professionals in the field and interested parties need access to data on military decision-making processes to understand where and how the scientific community is directing its investigations on the issue. Military decision-making is a strategic field of study because the military is crucial to the security and defense of a country or community. This work aims to be a point of reference for those involved in various capacities in military decision-making, providing key data regarding research trends over the years, the geographical distribution of scientific productivity, methodologies employed, annual statistics, and the prevalence of the most-investigated terms and topics. Therefore, this study serves as a bibliometric analysis of the literature on military decision-making publihed from 1992 to 2023 on the Scopus and Web of Science databases.

## 1. Introduction

### 1.1. Study of Decision-Making

Decision-making (DM) is a unique cognitive process that permeates many aspects of our lives. The study of DM spans several disciplines, from statistics to economics and from psychology to sociology. The expansion of this field of study extends to crucial contexts such as medical, political, economic, legal, organizational, business, and military decisions, as well as decision-making in emergency situations [1]. Therefore, understanding DM contributes significantly to navigating the challenges of life’s various and ever-changing contexts [2,3]. DM models are indispensable tools for understanding the principles and dynamics of DM. Studies of DM aim to gain a deeper understanding, influencing the design of solutions aimed at supporting decision-makers in making the most informed and accurate decisions possible, considering the information available at the time [2,4]. 

The study of DM has a rich history in psychology. It is necessary to refer to the succession of theoretical currents that, starting in the 1950s, extend to the present day, leading to the study of DM in the military context. The first theories in the field of DM were called normative theories [5]; they presupposed the human capacity to reason and represent problems and their solutions in a rational manner [6]. These theories were followed by descriptive theories [7]; they started from the assumption of the limited rationality of the human mind in representing problems and solutions [8]. Scott and Bruce described an individual DM style as the habitual response pattern of an individual in specific situations [9]. Interestingly, the authors emphasize that DM style is not a personality trait, but, rather, an acquired tendency to react in a particular way in certain DM contexts [10,11].

### 1.2. Military Decision-Making (MDM)

In the military, the process known as military decision-making (MDM) is an updated version of an analytical decision-making model originally adopted in 1960 and called “Situation Estimation”, a US Army doctrine known since 1910. This model originated from Prussian General Staff processes in the late 19th century, when it was developed to systematize military thinking and address the complexities and rigidities of warfare. In understanding how members of the armed forces make decisions in war, current military doctrine focuses on military decision-making (MDM)—an iterative process of identification, continuous assessment of threats, available resources, operational objectives, and selection of the best course of action [12,13]. The wealth of sometimes seemingly conflicting DM models highlights the inherent complexity of MDM [2].

The complexity of MDM manifests itself through several variables that are difficult to control. Complexity theory in the literature, derived from systems theory, takes the form of a diverse and heterogeneous subset underlying all innovation. This concept encompasses several and varied disciplines. Complex systems are characterized by self-organization and emerge from multiple and continuous interactions [14]. In complex systems, change results from the interaction between details and the variation in details resulting from each action [15]. This constant interaction between individuals, systems, and things creates a connection between actions and the environment, fueling innovation and evolution of the systems themselves [15]. The particularity of DM in this context is often characterized by a combination of high levels of uncertainty, precarious life-or-death conditions, strong time pressure, and the appearance of unpredictable real-life scenarios [7,10,16]. Generally, every decision tends towards a final choice or a series of choices resulting from an articulated evaluation process [17].

DM, the cornerstone for every military commander, serves as a bridge to translate the strategic vision into tangible actions [18]. This fusion requires commanders to apply analytical and practical approaches to solve problems of varying complexity. Complex contexts and environments require leaders to be highly adaptable and capable of formulating behavioral responses to meet diverse mission requirements; this capability may depend on their competence to facilitate effectiveness in a wide range of roles [10,19,20]. History tends to associate successes and failures with the quality of the situation assessment and decisions made by the military commander in preparing and executing operations [21]. The key issues that a commander and his staff face during the planning of operations concern decisions on the definition of the operation and the determination of the method of execution. To make these decisions, the command must understand the intention and objectives of the higher command regarding the specific task. While secondary processes may occur simultaneously, the core of the planning and its main results lie in the definition of the task and how it is to be accomplished. The mission is defined by the commander based on an order or directive from the higher command or on their own initiative, given their understanding of the situation and their assigned responsibility.

Deciding how to use force to fulfill a mission is an expression of the commander’s military leadership. To execute DM, the commander must gain a deep understanding of operational issues and formulate solutions that achieve mission objectives as efficiently and effectively as possible. A strategic process in MDM is that of Command and Control (C2) applications. Command and Control operations are how a commander issues orders and directives and ensures that they are respected and properly implemented [22]. The requirements for a C2 system that can provide satisfactory responses and actions are as follows: constant monitoring and verification of information by the command, coordination of communications, and actions to be delegated to lower hierarchical levels. New technologies offer opportunities to improve and extend all the activities of those exercising command and control of operations in the military [23,24,25]. However, these supports cannot ignore the essential role of the human being in DM [26]. MDM is also a learning process in which lessons from past events form a basis for optimizing future decisions. According to Argyris and Schön [27], it is imperative that military organizations demonstrate the ability to adapt and assimilate lessons derived from mistakes made to enhance their performance in future engagements. Military doctrine attempts to provide a DM process for operations planning to generate these two products, i.e., defining the mission and defining the method, along with other aspects required by command, from receiving operational tasks from superiors to assigning operational tasks to subordinates. The DM process is generally presented as a model consisting of steps and results. 

A direct continuation of the DM process during planning is the operational command and control process, but this is beyond the scope of this essay [21]. For the military commander, improving DM capabilities is not just a matter of professionalism; it can be a matter of life and death [19]. Psychological factors influence people’s perceptions and preference for risk, which can affect the quality of their decisions [28]. As stated by Sun Tzu in the treatise entitled “The Art of War”: “Know the enemy, know yourself, and you will not fear the outcome of a hundred battles” [29].

### 1.3. Aim of the Study

Considering the existing literature, we believe that this study could be an innovative and comprehensive reference point on scientific productivity in the field of MDM, given the current lack of similar studies and thus the gap in the literature. Having data available in an essential sector for governments, especially considering the rapid pace of contemporary geopolitical developments, is of strategic importance and is also a valuable support for scholars and researchers. The research question that inspired this review was, “What are the trends in scientific publications in the field of MDM over the past thirty years?” Based on this premise, this article will examine the trends from the last three decades in the literature related to MDM.

The analysis will identify the main journals used for publications, the main types of articles published, the most productive countries, the most frequently used language, and the key figures that have significantly contributed to the scientific production on the subject. It will also trace the publication trends over the years (number of publications per year) from the beginning of 1992 to the end of November 2023. It will also provide a ranking of the top authors with the greatest number of citations and co-authorships and a ranking of the top ten most-cited articles included in this bibliometric analysis. Bibliometric analysis is a well-established and meticulous methodology for exploring and examining a broad corpus of publications and scientific data [30,31,32,33,34]. Through it, it is possible to capture the subtle evolutionary nuances of a specific research field while illuminating new and emerging areas in that field.

It also enables the identification of emerging trends in article and journal performance, the elucidation of collaboration patterns and key elements of research, and the exploration of the intellectual structure of a particular field in the existing literature. This approach facilitates knowledge mapping, stimulates innovation, and enables researchers to identify gaps, generate new ideas, and enhance their contributions in the field. This review, a unique and innovative contribution to the field of MDM, aims to highlight key resources and, consequently, to assist researchers in this specific field in finding material more easily to start their studies, to have a clearer idea of what already exists in the literature, and to guide further research. This study proposes two hypotheses: the first started from the assumption that with the outbreak of a major conflict, there should be an increase in scientific productivity in the field of MDM (H1), and the second was based on the assumption that one or more world military superpowers would have greater scientific productivity in this sector (H2). This study did not require ethical approval as it did not involve human or animal subjects and as the research was conducted using data available in the public domain. The article is structured as follows: In Section 1 (Introduction), which consists of three subsections, the theoretical framework with the main reference theories, the research question, and the formulated hypotheses are presented (Section 1.1 Study of DM, Section 1.2 Military decision-making (MDM); Section 1.3 Aim of the study); Section 2 (Materials and Methods), which consists of three subsections (Section 2.1 Design of the study, Section 2.2 Data extraction, Section 2.3 Data analysis), describes the tools and methodology used; Section 3 (Results), which consists of six subsections (Section 3.1 Type of articles and language, Section 3.2 Analysis of leading countries, Section 3.3 Analysis of leading journals, Section 3.4 Analysis of authors and co-cited authors, Section 3.5 Analysis of co-occurring keywords, Section 3.6 Top ten articles cited), is where the results will be presented; Section 4 (Discussion) is where the discussions will be addressed in light of the results obtained and the literature covered in the introduction; Section 5 (Conclusions) is where future development areas will be outlined in light of the results presented and discussed in this research.

## 2. Materials and Methods

### 2.1. Design of the Study

The bibliometric analysis was conducted on publications from two scientific databases: Scopus and Web of Science. The selection of the most relevant documents was guided by specific inclusion and exclusion criteria, ensuring a rigorous and well-defined process. Both databases enjoy a solid reputation in the field of bibliometric research, as evidenced by numerous studies. Scopus and Web of Science boast an extensive repertoire of high-quality international academic publications, which is key to ensuring the validity and representativeness of the analysis conducted. Moreover, both offer comprehensive and reliable metadata suitable for conducting in-depth bibliometric analyses, as demonstrated by previous research. A further advantage offered by Scopus and Web of Science is their extended temporal coverage, which enables exploration of the evolution of publications over time and identification of significant trends in the field of study under consideration. Such a feature is crucial for capturing the development and impact of research in historical context and tracing the path of knowledge over time. To select articles for inclusion, a search was conducted using the following string: “(Military decision-making)”. This query returned 390 records, specifically 144 on Scopus and 246 on Web of Science. The search covered the years from the beginning of 1992 to the end of November 2023.

### 2.2. Data Extraction

Two reviewers independently processed the review of the titles and abstracts of the selected articles. Fifty articles were analyzed at a time. Any discrepancies were resolved through comparison between the two reviewers, aiming to reach a consensus. Publications belonging to categories such as theses, books, or other forms of publications apart from scientific articles were excluded. Research related to fields and topics like MDM was also excluded. At the conclusion of the article-selection process, data extraction took place. The data extracted from each publication included the following: citation information (authors, paper title, year, source title, volume, numbers, citation count, and DOI); bibliographic information (affiliation, publisher, and language of the original paper); abstracts; author keywords; indexed keywords; and funding details (funding number, sponsor, and text). These data were imported into an Excel spreadsheet for further processing.

### 2.3. Data Analysis

Co-citation and co-occurrence analyses were conducted using VOSviewer version 1.6.20, developed by N. J. Eck and L. Waltman, which provides a graphical representation resulting from multivariate statistical techniques [35,36,37]. Analyses on frequency distributions of extracted data regarding article type, language used, geographical distribution, main journals, and main authors of the identified publications were conducted using Microsoft Excel version 2404. To map and monitor the status of research on MDM, a preliminary analysis was conducted based on year of publication, article type, and language of publication. The citation count highlights the most relevant works among those included. Bibliometric analyses such as co-authorship analysis and co-occurrence analysis were performed. Co-authorship analysis is a technique that highlights intellectual cooperation in scientific research and involves the participation of two or more authors from the same geographical context or from different areas in the conception and writing of a study [38]. Co-occurrence analysis is a textual analysis aimed at identifying the keywords that are most frequently repeated in the title and abstract of the included scientific publications. VOSviewer provides graphical outputs summarizing the conducted analyses. Each VOSviewer graph consists of nodes or labels, the size of which represent the number of documents found in the literature, and arcs, which represent the relationships between authors, countries, or keywords. Moreover, the software allows, using multivariate statistics, the creation of clusters in relation to the object of the analysis technique (keyword clusters, country clusters, author clusters, etc.).

## 3. Results

**Figure 1:**
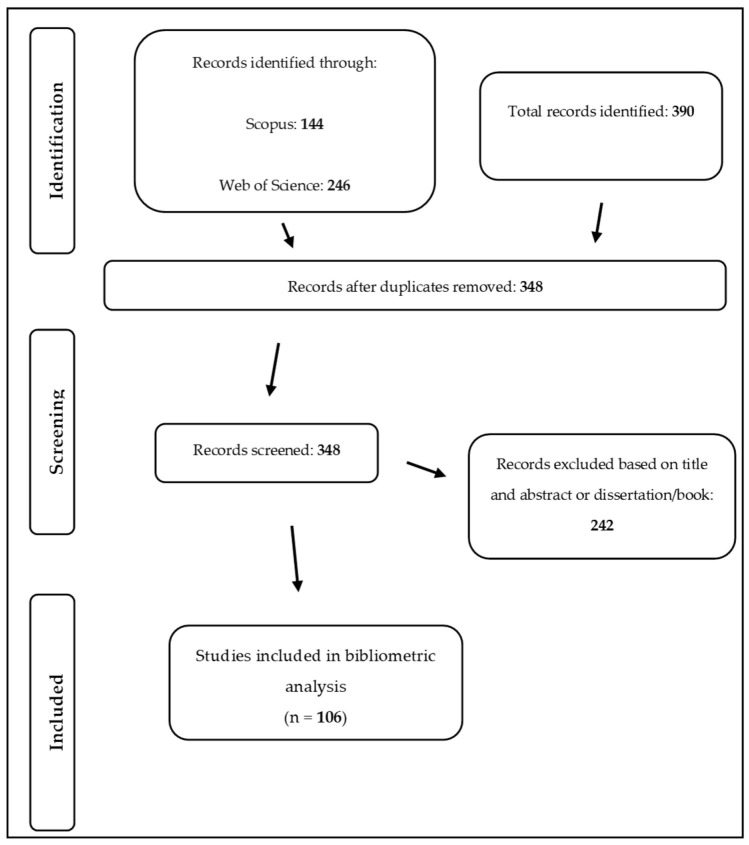
Article-selection process.

**Figure 2:**
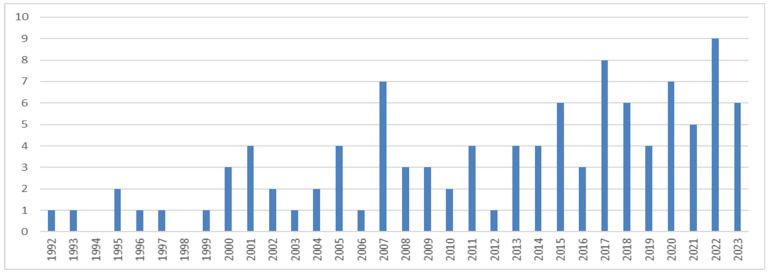
Number of articles focused on MDM published between 1992 and November 2023 (data collected on Scopus and Web of Science).

The search strategy produced 390 articles. Of these, 42 were duplicates and were therefore excluded. A further 242 records were excluded after application of the eligibility criteria regarding the title and abstract. The full text of the 106 articles represented the core of the scientific literature on DM in emergency situations. Figure 1 summarizes the steps that led to the identification of the selected articles. From the beginning of 1992 to the end of November 2023, a total of 366 authors contributed to the literature (Figure 2). The papers were mainly co-written, with multi-authored papers accounting for 82% of the corpus. The oldest publication among those examined was an article by Rose McDermott entitled ‘Prospect theory in international relations: The Iranian hostage rescue mission’, which was published in English in Political Psychology in 1992 [39]. 

As shown in detail in Figure 2 and summarized in Table 1, although the discussion of military decision-making dates to earlier times (the first article retrieved dates to 1992), there has been a significant increase in publications on the topic in the decade from 2013 to 2023. During these years (2013–2023), 58.5% of the total scientific corpus included in the bibliometric analysis was published. The clear fact is that there has been a significant increase in scientific productivity on the topic from 2007 onwards.

### 3.1. Type of Articles and Language

The sample of publications subjected to bibliometric analysis consisted mainly of original articles (94.4%), preview articles (4.7%), and conference proceedings (0.9%) (Table 2), predominantly in English (98.2%) (Table 3).

### 3.2. Analysis of Leading Countries

**Figure 3:**
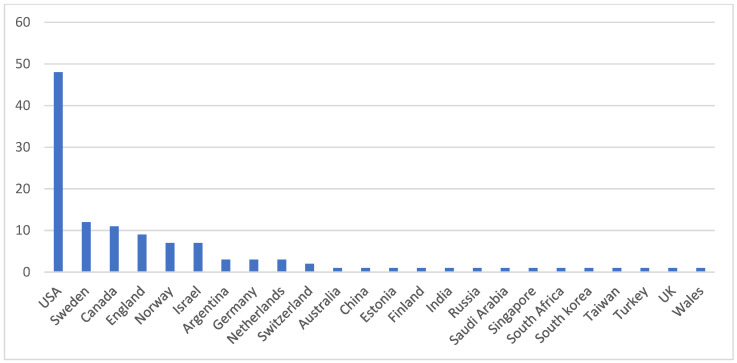
Graphical representation of the most productive countries for scientific articles.

The studies included in this bibliometric analysis were published by researchers in 24 different countries. The top 10 countries contributing to research on DM in emergency situations are the United States, Sweden, Canada, England, Norway, Israel, Argentina, Germany, the Netherlands, and Switzerland (Table 4). The three countries with the greatest number of citations are the United States (914), Sweden (408), and Canada (133). The United States of America is the most productive country, as can be seen from the graph in Figure 3, with four times as many articles as Sweden.

### 3.3. Analysis of Leading Journals

Analyzing publication sources is a useful way to identify the main journals within a specific research area. This is crucial for researchers searching for articles and identifying the most suitable journal for their work. Regarding the results of the literature search, Table 5 presents the main publication sources in the field of MDM. Furthermore, Table 5 lists all publication sources with at least three published articles on this topic. It should be noted that “Military Psychology” emerges as the most prolific publication source, with twenty articles, followed by “Human Factors”, with fourteen articles.

The impact factor (I.F.) of each cited journal was also reported, having been obtained by dividing the total number of citations of each article published in the journal during a given period by the total number of articles published in that same journal during the same period. The result represents the average number of citations received per article published in the journal during the given period. The impact factor of a scientific journal is a metric used to assess the relevance and influence of the journal within the scientific community [40].

As far as the citation analysis is concerned, it can be seen that “Human Factors” is the journal with the greatest number of citations among the articles included in this bibliometric analysis, with 494 citations. This journal is followed by “Ergonomics”, which has 170 citations among the five articles included (Table 5).

### 3.4. Analysis of Authors and Co-Cited Authors

The authors who have written most on the topic of MDM in the reference years and who can be considered experts in the field were identified through co-authorship network analysis. In total, 366 authors are associated with the articles included in this bibliometric analysis on MDM. Table 6 provides the list of the most significant authors in terms of the number of publications included. Biggs A.T. stands out as the most prolific author, with six articles [41,42,43,44,45,46], followed by Thunholm P., with five articles [10,11,47], Azzolini S.C. [48,49,50], Hamilton J.A. [42,44,46] and Thompson M.M. [51,52,53], with three articles. Furthermore, considering the total number of citations received, Thunholm P. leads the way with 258 citations in total, followed by Thompson M.M., with 62 citations (Table 6).

**Figure 4:**
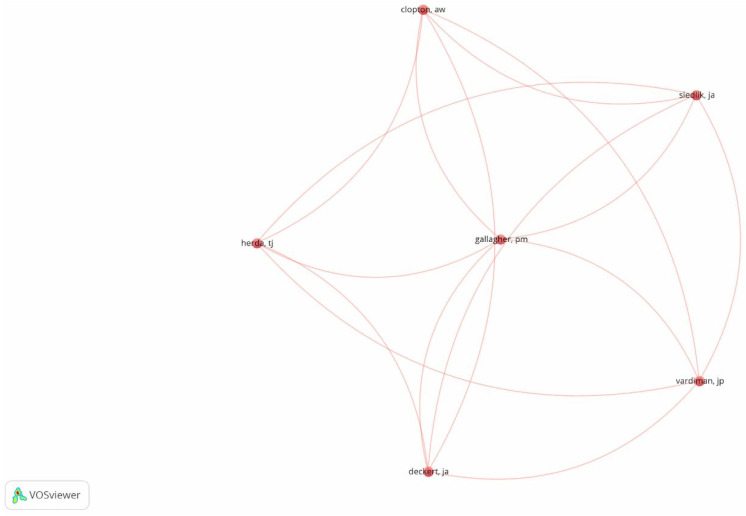
Co-citation, cited authors, minimum number of citations of an author: 2. Most interconnected authors.

We conducted a co-citation analysis of the cited authors, using the 106 articles selected at the end of the article-screening process for review as the unit of analysis. For this purpose, we used VOSviewer software. We created a library in RIS format and uploaded it to VOSviewer to proceed with the analysis; subsequently, we selected “full counting” mode for the most frequently recurring authors. The unit of analysis is the author. A citation analysis was conducted based on the authors cited, setting a minimum number of citations of two authors per document. The relevance criterion was at least 60% occurrence among the selected articles. This process led to the selection of the 69 most frequently co-cited authors among the selected articles. However, not all of these authors were connected to each other. The strength-association method was used to normalize the strength of the connections between the items. Apart from a multiplicative constant, this method is identical to Equation (6) in van Eck and Waltman [54]. A summary map of the process described above can be seen in Figure 4.

### 3.5. Analysis of Co-Occurring Keywords

**Figure 5:**
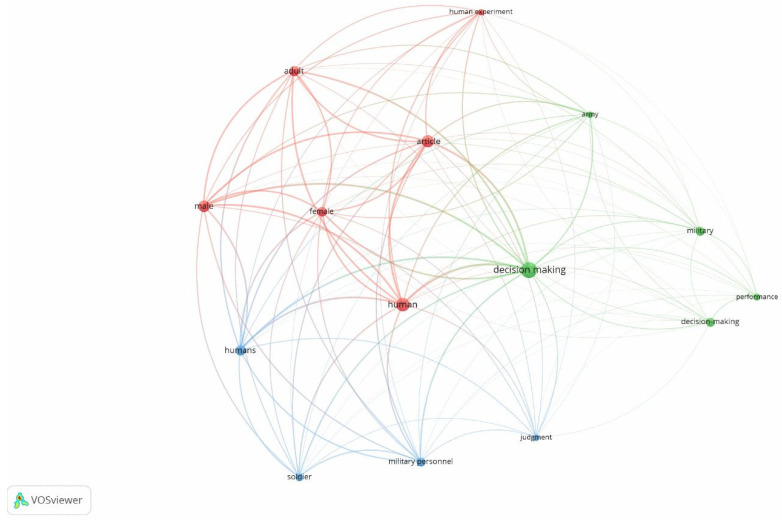
Co-occurrence analysis on the topic of MDM. The units of analysis were index keywords, with a total of 2854 index keywords identified. The analysis employed a binary count with a minimum occurrence threshold set at 30. In total, 15 keywords were identified.

Figure 5 shows a co-occurrence analysis on the topic of MDM. The units of analysis were index keywords, with a total of 2854 index keywords identified. The analysis employed a binary count with a minimum occurrence threshold set at 30. In total, 15 keywords were identified (decision making, human, article, male, adult, female, humans, military, decision-making, soldier, military personnel, judgement, performance, and army). The size of the nodes represents the number of keywords found in the literature that were most frequently encountered, and the arcs represent the relationships between the keywords; the colors of the nodes and arcs represent the clusters of the keywords. The analysis revealed the existence of three clusters of keywords: “sample variables” in red (adult, human experiment, article, female, human, and male); “decisions” in green (decision making, decision-making, military, performance and army); and “human” in blue (humans, military personnel, soldier, and judgement).

Some terms were excluded because they were considered insignificant for representation purposes (statistics, numerical data, major clinical study(s), priority review, middle-aged, elderly, prospective study(s), controlled study(s), child, retrospective study(s), observational study(s), and questionnaire).

### 3.6. Top Ten Articles Cited

Table 7 presents the characteristics of the ten most-cited articles on MDM in the world. The ten most-cited articles received between 133 and 46 citations [10,11,19,20,23,24,25,39,55,56].

## 4. Discussion

This search resulted in the identification of 106 articles extracted from two major scientific databases that explore the topic of decision-making in military settings. Numerous studies have explored MDM in emergency contexts during the period from 1992 to 2023 (November). Various bibliometric analyses, including frequency tables, percentage values, and scientometric indices, along with co-occurrence analyses, were performed to quantitatively assess the impact and relevance of the topic of MDM in the scientific field. The analysis revealed a steady increase in published research from 2007 onwards, with scientific articles being the most-observed type of work, followed by preview articles. English emerged as the predominant language in these publications. The United States and Sweden showed the highest productivity in terms of published work, followed by Canada. Keyword analysis, including index terms, titles, abstracts, and authors, produced noteworthy results, highlighting connections with terms related to topics and fields associated with MDM (decision making, human, article, male, adult, female, humans, military, decision-making, soldier, military personnel, judgment, performance, and army). From the ten most-cited articles, it emerged that it is imperative to conduct research in the field of MDM while considering the variables of human psychology that remain connected to the world of DM despite the strong institutionalization and hierarchization of decisions in this area. Considering the reported results, we believe we have addressed the initial research question, namely, “What are the trends of scientific publications in the MDM sector over the last thirty years?” and consider both hypotheses verified. Indeed, the first hypothesis posited the assumption that with the outbreak of a major conflict, there would be an increase in scientific productivity in the MDM sector (H1). Looking at Figure 2, it can be noted that the years with the highest scientific productivity are 2007, 2017, and 2022. Moreover, as shown in Table 1, where scientific productions were divided among the three decades of the period under consideration, the value of the data representing scientific productivity for the last decade (2012–2023) is noticeably higher compared to those from the other two preceding decades. Confirmation of this hypothesis stems from a listing of the main conflicts that erupted from 2007 to 2023, including the intensification of the Israeli-Palestinian conflict starting from 2008 [57], the outbreak of the “Arab Spring” in 2011 [58], the onset of the war in Syria in 2011 [59], the eruption of the conflict in Yemen in 2014 [60], the conflict in Afghanistan lasting from 2001 to 2021 [61], the outbreak of the Russo-Ukrainian conflict in 2022 [62], and the exacerbation of the Gaza conflict in 2023 [63], just to name a few without forgetting all the other conflicts worldwide. The second hypothesis envisaged that one or more world military superpowers would exhibit greater scientific productivity in the MDM sector (H2), and indeed, at the top of the list of the most scientifically productive countries is the United States of America, as shown in Table 4. The main limitation of this study is that it did not consider more scientific databases; however, this limitation can be re-evaluated if one considers that two of the main scientific databases at a global level were consulted. This bias can be addressed by replicating the study and utilizing all the remaining databases not used in this study. Another limitation is related to the inclusion criteria adopted and to the search string used. Again, we hope that this study will be replicated and further improved for a more comprehensive treatment of the topic addressed.

## 5. Conclusions

DM in the military context is a sensitive topic that ranges from simple decisions made by instructors during training and in routine contexts to rapid decisions made by commanders in conflict and war situations. The array of variables that characterize DM in the military context is diverse and often involves the unpredictability of intervening events and time pressure, making the decisions themselves complex. Complex-systems theory offers an excellent key to understanding and studying DM in the military context. Making decisions as part of the daily routine is different from making decisions in the military context, sometimes in critical scenarios. Tracing the path of scientific publications in the field of military decisions over the past three decades was the aim of this article, which provides bibliometric data. This work will serve professionals in the field as an analytical tool through which to observe research trends and identify strengths and weaknesses in scientific productivity within the MDM field. This study demonstrates its originality, as it is the only one in the literature that maps the bibliometric and scientometric trends in the field of MDM studies. We hope that this research will inspire experts in the MDM field to inquire in the future about the reasons behind certain statistical trends and thus formulate more specific research questions based on the results presented in this article, above all by using other scientific databases. Additionally, this work can be extremely useful for the defense sectors of various governments, providing historically traced data that can allow them make to comparisons or establish future lines of development and research in the MDM sector.

## Figures and Tables

**Table 1:** Analysis of publications by year.

Years	n. of Articles	% of 106
From 1992 to 2001	14	13.2
From 2002 to 2011	29	27.4
From 2012 to 2023 *	63	59.4
Total number of articles included	106	

The recovery of scientific studies was conducted in November 2023. Therefore, the number of articles and its corresponding percentage correspond to the count and the count divided by the total number of decades, respectively. * With reference to the last count, we considered data up to November 2023.

**Table 2:** Analysis on the type of publication.

Document Type	n. of Articles	% of 106
Article	100	94.4
Article; Early Access	5	4.7
Article; Proceedings Paper	1	0.9
Total	106	

**Table 3:** Languages of publications.

Language of Original Document	n. of Articles	% of 106
English	104	98.2
Russian	1	0.9
Spanish	1	0.9
Total	106	

**Table 4:** Table representing the number of articles detected per country and the number of citations per country, extracted from the database.

Country	n. of Articles	Citations
United States of America	48	914
Sweden	12	408
Canada	11	133
England	9	78
Norway	7	78
Israel	7	66
Argentina	3	86
Germany	3	64
Netherlands	3	9
Switzerland	2	28
Australia	1	0
China	1	1
Estonia	1	1
Finland	1	9
India	1	10
Russia	1	2
Saudi Arabia	1	30
Singapore	1	12
South Africa	1	19
South Korea	1	6
Taiwan	1	0
Turkey	1	6
United Kingdom	1	13
Wales	1	4

**Table 5:** List of journals published on MDM.

Journal Name	Total Papers	% of 106	I.F. *	Total Citation
Military Psychology	20	18.8	1.27	132
Human Factors	14	13.2	3.59	494
Frontiers in Psychology	5	4.7	4.23	20
Ergonomics	5	4.7	2.56	170
Ethics & Behavior	4	3.8	2.14	34
Journal of Cognitive Engineering and Decision Making	4	3.8	0.58	24
Scandinavian Journal of Psychology	4	3.8	2.31	108
Applied Ergonomics	3	2.8	3.94	13
Personality and individual Differences	3	2.8	3.95	153

* Impact Factor (I.F.): Impact factor data were retrieved from Academic Accelerator (https://academic-accelerator.com/) and refer to the years 2022–2023. Data retrieved on 7 December 2023.

**Table 6:** List of the most significant authors in terms of number of publications and number of citations included in this bibliometric analysis.

Author	n. of Articles	n. of Citations
Biggs A.T.	6	52
Thunholm P.	5	258
Azzollini S.C.	3	9
Hamilton J.A.	3	26
Thompson M.M.	3	62

**Table 7:** List of the ten most-cited articles about MDM in the world from 1992 to November 2023.

Authors	Title	Publication Year	Journal	n. Citation *	Quartile
[10]	Decision-making style: habit, style, or both?	2004	Personality and Individual Differences	133	Q1 (Psychology)
[25]	Developing operator capacity estimates for supervisory control of autonomous vehicles	2007	Human Factors	109	Q1 (Applied Psychology)
[20]	The Psychological and Neurological Bases of Leader Self-Complexity and Effects on Adaptive Decision-Making	2013	Journal of Applied Psychology	108	Q1 (Applied Psychology)
[55]	The measurement of team process	1995	Human Factors	101	/
[56]	Team situation assessment and information distribution	2000	Ergonomics	81	Q2 (Human Factors and Ergonomics)
[39]	Prospect theory in international relations: The Iranian hostage rescue mission.	1992	Political Psychology	54	/
[19]	Individual differences in shifting decision criterion: A recognition memory study	2012	Memory & Cognition	50	Q1 (Experimental and Cognitive Psychology)
[11]	Decision-making styles and physiological correlates of negative stress: Is there a relation? Cognition and Neurosciences	2008	Scandinavian Journal of Psychology	48	Q2 (Psychology)
[23]	Heuristic automation for decluttering tactical displays	2005	Human Factors	46	Q2 (Applied Psychology)
[24]	Effects of automation of information-processing functions on teamwork	2005	Human Factors	46	Q2 (Applied Psychology)

## Data Availability

The data from this research can be requested at the following e-mail address: ivan.dalessio@uniroma1.it.
